# *Choanephora infundibulifera* Rhinosinusitis in Man with Acute Lymphoblastic Leukemia, Tennessee, USA

**DOI:** 10.3201/eid3006.230794

**Published:** 2024-06

**Authors:** Anita Max, Heather L. Glasgow, Teresa C.B. Santiago, Ashley Holland, Hiroto Inaba, Connie F. Cañete-Gibas, Nathan P. Wiederhold, Randall T. Hayden, Elisabeth E. Adderson

**Affiliations:** St. Jude Children’s Research Hospital, Memphis, Tennessee, USA (A. Max, H.L. Glasgow, T.C.B. Santiago, A. Holland, H. Inaba, E.E. Adderson);; University of Tennessee Health Sciences Center, Memphis (H. Inaba, E.E. Adderson);; University of Texas Health Science Center, San Antonio, Texas, USA (C.F. Cañete-Gibas, N.P. Wiederhold)

**Keywords:** *Choanephora infundibulifera*, rhinosinusitis, lymphoblastic leukemia, Tennessee, United States, fungi

## Abstract

*Choanephora infundibulifera* is a member of the Mucorales order of fungi. The species is associated with plants as a saprophyte or parasite and may be responsible for spoilage or disease but is an uncommon cause of human infection. We describe *C. infundibulifera* rhinosinusitis in a young man with leukemia in Tennessee, USA.

An 18-year-old man visited St. Jude Children Research Hospital in Memphis, Tennessee, USA, with systemic symptoms and lymphadenopathy and received a diagnosis of early T-cell precursor acute lymphoblastic leukemia. Induction chemotherapy was complicated by rhinosinusitis linked to species of *Alternaria* and *Curvularia* and presumed fungal pneumonia. The man’s treatment consisted of debridement of his nasal and sinus passages and administration of liposomal amphotericin B, followed by oral posaconazole for 5 months. Thereafter, posaconazole secondary prophylaxis was prescribed during severe neutropenia.

The man’s leukemia relapsed 6 months after his original diagnosis, and he received treatment that included cyclophosphamide, vincristine, doxorubicin, methotrexate, cytarabine, dexamethasone, dasatinib, and venetoclax. At 4 months postrelapse, while receiving posaconazole prophylaxis (300 mg orally 2×/d), the patient sought medical treatment for acute right facial pain and a black eschar on his anterior nasal septum. His leukocyte count was 0.16 × 10^3^ cells/mm^3^ (normal 4.5 to 11.0 x 10^3^ cells/mm^3^), and his absolute neutrophil count was 20 cells/mm^3^ (normal 1,500 to 8,000 cells/mm^3^). Measurement of his serum posaconazole trough concentration revealed a level of 1.4 μg/mL (desired concentration ≥0.7 μg/mL). Computed tomography of the sinuses showed evidence of rhinosinusitis (Figure 1, panel A). A magnetic resonance imaging scan revealed soft tissue swelling, right nodularity and irregular nasal septal mucosal thinning, sinus mucosal thickening, and enhancing right jugular lymph nodes. Computed tomography of the chest yielded unremarkable results. The patient used smokeless chewing tobacco and electronic cigarettes but had an otherwise unremarkable exposure history.

**Figure 1:**
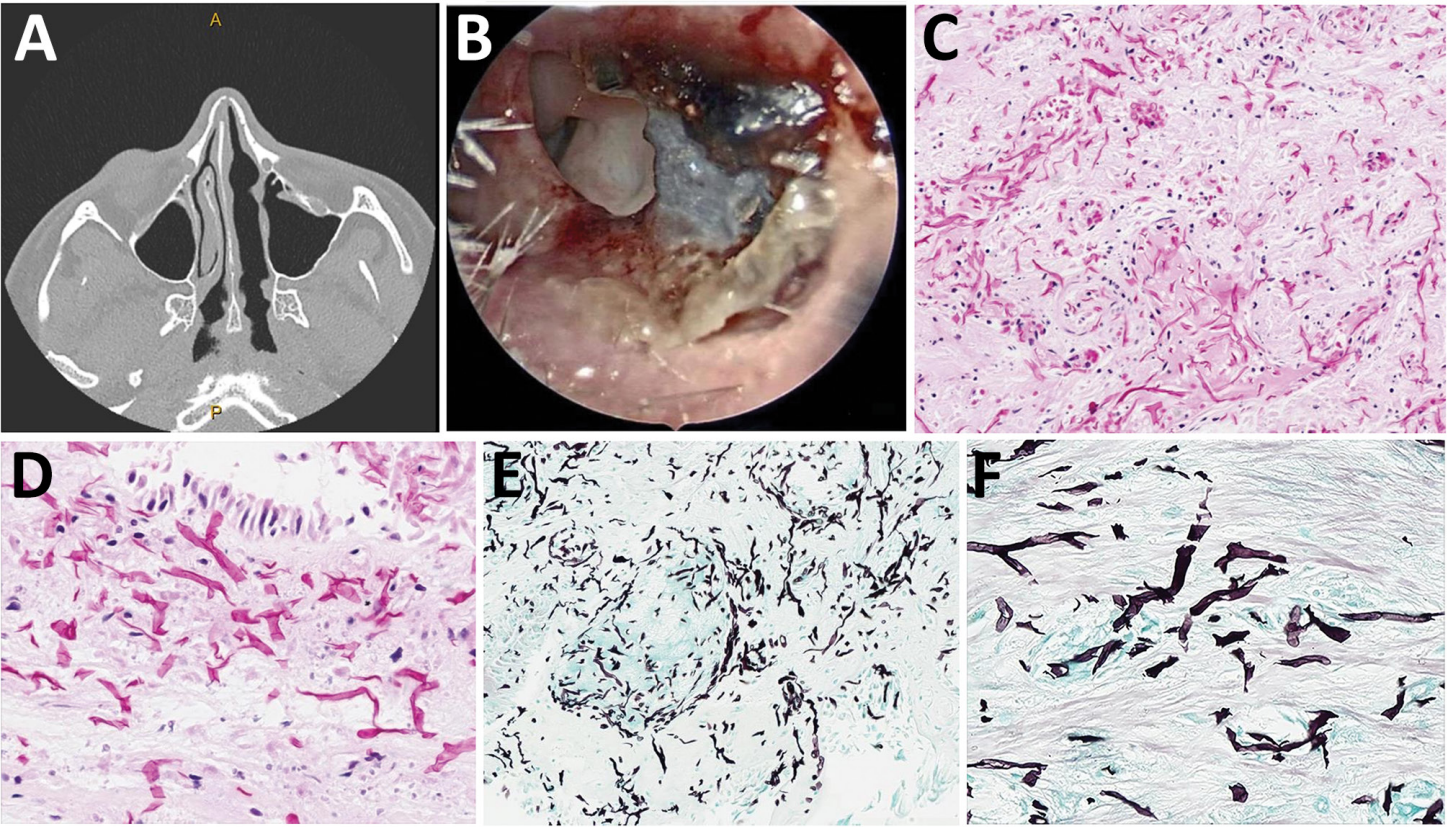
Computed tomography, endoscopic findings, and histomorphology of rhinosinusitis caused by *Choanephora infundibulifera* in a man with leukemia in Memphis, Tennessee, USA*.* A) Computed tomography shows new asymmetric swelling of the anterior nasal septum and irregularity of the right septum, edema of the inferior turbinates, and obstruction of the right frontal sinus outflow tract. A septal perforation, the sequela of the patient’s previous fungal rhinosinusitis, was stable. B) Nasal endoscopy reveals necrosis of the anterior nasal septa. C, D) Necrotic sinonasal mucosa contains numerous hyaline (nonpigmented) fungal elements with broad (ribbon-like), thin-walled, nonseptated, and pleomorphic fungal hyphae. Hematoxylin and eosin stain; original magnification ×200 for panel C, ×400 for panel D. E, F) Gomori methenamine-silver stain highlights the fungal elements (in black). Original magnification ×200 for panel E, ×400 for panel F.

The patient underwent nasal endoscopy and debridement (Figure 1, panel B). Hematoxylin and eosin–stained sections from a biopsy of the right nasal septum revealed necrotic tissue with numerous hyaline fungal elements with a wide. ribbon-like appearance, further highlighted by Gomori methenamine-silver staining (Figure 1, panels C–F). Technicians isolated coagulase-negative *Staphylococcus* and *Enterococcus faecium* from bacterial cultures but considered those elements contaminants. We obtained 2 isolates from a fungal culture, identifying 1 microscopically, on lactophenol cotton blue stain, as a *Curvularia* species. Further testing by matrix-assisted laser desorption/ionization time-of-flight mass spectrometry (Vitek MS V3.2; bioMérieux, https://www.biomerieux.com) revealed the isolate to be *Curvularia lunata*. We determined the other isolate to be *Choanephora infundibulifera* by phenotypic characterization (Figure 2) and BLAST searches (https://blast.ncbi.nlm.nih.gov/) using sequences of the nuclear ribosomal internal transcribed spacer region (GenBank accession no. OR643928) and the D1 and D2 domains of the 28S rRNA gene (GenBank accession no. OR643927). BLAST search results matched with reference strains as follows: internal transcribed spacer region, *C. infundibulifera* CBS 153.51 99.46%, *C. infundibulifera* KUS-F27535 99.49%; D1 and D2 domains of the 28S rRNA gene, *C. infundibulifera* CBS 153.51 100%, *C. infundibulifera* KUS-F27535 100% (*1*–*3*). We determined MICs for amphotericin B (≤0.3 μg/mL), micafungin (>8 μg/mL), voriconazole (>16 μg/mL), posaconazole (1 μg/mL), and isavuconazole (>16 μg/mL) per the Clinical and Laboratory Standards Institute’s broth microdilution method (*4*).

**Figure 2:**
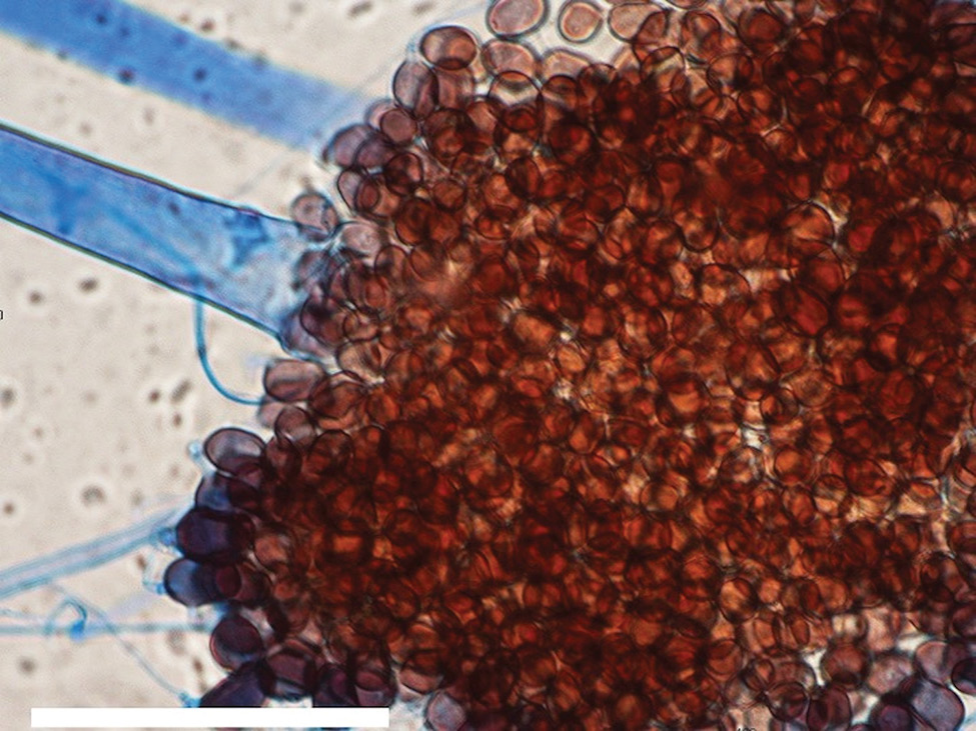
Microscopic morphology of *Choanephora infundibulifera* showing sporangiophore-bearing sporangiola and sporangiospores from a tape mount on banana leaf agar for 7 days at 25°C. Scale bar indicates 100 µm.

We initiated liposomal amphotericin B (5 mg/kg/d) as treatment and continued posaconazole. We directed the patient to receive 4 additional nasal and sinus debridements over 2 weeks, observing fungal elements in biopsies obtained from the first 3 operations. Cultures from all biopsies, however, were sterile. We transitioned the patient to amphotericin B (3×/wk) after 3 weeks and discontinued posaconazole 1 month thereafter. The patient’s nasal pain and tenderness progressively improved. Otolaryngology evaluation 4 weeks after the onset of infection was unremarkable. Unfortunately, the patient died of refractory leukemia 4 months after the diagnosis of his fungal infection (14 months after leukemia diagnosis).

## Conclusions

The Mucorales group consists of over 260 species in 55 genera that are ubiquitous in wet, organic environments. Approximately 40 species are clinically significant, causing invasive infection (mucormycosis) chiefly in persons with diabetes and immunocompromising conditions (*1*). The genus *Choanephora* (family *Choanephoraceae*) contains 2 species, *C. infundibulifera* and *C. cucurbitarum* (*5*). These species are saprophytes or parasites of plants that can promote spoilage and disease (*6*). *C. cucurbitarum*, the more commonly recognized species, causes wet blight, flower rot blight, and leaf blight, chiefly on summer squash and other cucurbits (*7*).

First described by Currey in 1873, *C. infundibulifera* infrequently causes plant disease but has been implicated in twig and leaf rot and blossom blight (*8*–*11*). On potato-carrot or potato dextrose agar, colonies grow rapidly at 25°C with abundant white, pale-yellow, or brown mycelia and sporangiophores, with sporangia arising from substrate mycelium or nonseptate, unbranched, hyaline aerial hyphae (*11*). Definitive identification is based on morphology and sequencing of the nuclear ribosomal internal transcribed spacer region and the D1 and D2 domains of the 28S rRNA gene.

In the patient we describe, *Curvularia* species was among those isolated from the initial nasal biopsy, but histopathologic features observed in multiple biopsies over 2 weeks suggested that this was not the predominant pathogen. The fungal elements we observed in the infected tissue were consistently suggestive of an infection caused by a species in the order of Mucorales rather than a species of *Curvularia*, a dematiaceous mold that is typically pigmented, with septate and often acutely branched hyphae. Furthermore, the patient’s clinical course, with progressive tissue necrosis necessitating serial debridement to achieve a cure, was more consistent with the aggressive disease characteristic of a species in the order of Mucorales (*12*).

We could not determine a clear source of the patient’s fungal infection. We noted that he had limited exposure to the outdoors in the weeks before his infection and no close contact with plants or soil. We did not obtain hospital and domiciliary environmental samples; however, we did determine that no additional cases of infection caused by *Choanephora* or *Curvularia* species were reported in the hospital proximate to the patient’s illness.

The optimal treatment for infections caused by *Choanephora* species is unknown. The minimal inhibitory concentration correlation with treatment response in vivo is unknown, but the in vitro antifungal minimal inhibitory concentrations against this isolate suggest amphotericin B might have greater activity than posaconazole and isavuconazole, which are used to treat mucormycosis caused by other species. Consistent with the antifungal susceptibility results, our patient’s infection developed while he was receiving secondary prophylaxis with posaconazole. Treatment with liposomal amphotericin, initially in combination with posaconazole and with adjunctive surgical debridement, led to clinical and microbiological resolution of his infection despite ongoing cancer therapy and neutropenia. This report of human nasal infection caused by a species of *Choanephora* serves as a reminder that emerging fungal pathogens continue to pose clinical challenges, especially in severely immunocompromised patients.
